# Advanced undergraduate medical students’ perceptions of basic medical competences and specific competences for different medical specialties – a qualitative study

**DOI:** 10.1186/s12909-022-03606-1

**Published:** 2022-08-01

**Authors:** Elena Zelesniack, Viktor Oubaid, Sigrid Harendza

**Affiliations:** 1 III. Department of Internal Medicine, University Medical Centre Hamburg-Eppendorf, Hamburg, Germany; 2 German Aerospace Centre (DLR), Hamburg, Germany

**Keywords:** Competence, Medical specialty, Medical student, Residency, Postgraduate medical education, Undergraduate medical education

## Abstract

**Background:**

Medical graduates should have acquired basic competences that enable them to practice medicine independently as physicians and to enter postgraduate training in any specialty they wish. Little is known about advanced undergraduate medical students' perceptions of basic medical competences needed to start postgraduate training and about specialty-specific competences. This qualitative study aims to identify medical students’ perceptions of basic medical competences and specific competence requirements for different specialties.

**Methods:**

In December 2020, sixty-four advanced undergraduate medical students participated in the role of a resident in a competence-based telemedicine training simulating a first day in postgraduate training. After the training, eight focus group interviews were conducted about students’ perceptions of basic medical competences and specialty-specific competences using a semi-structured interview guide. The interviews were transcribed and analysed thematically according to the six steps of Braun and Clarke. The analysis was carried out by an inductive search for themes, which were deductively assigned to the six competence areas of the requirement-tracking questionnaire (R-Track).

**Results:**

Regarding basic medical competences, four R-Track competence areas could be identified as main themes. The students considered ‘*Social-interactive competences’* to be particularly relevant for basic clinical work, including ‘*Structuring information’*, ‘*Tactfulness*’, and ‘*Stress resistance’*. Students especially emphasized ‘*Concentration*’ as an important aspect of the competence area ‘*Mental abilities*’. Among ‘*Personality traits*’, ‘*Honesty*’ was mentioned most frequently, and students were also aware that ‘*Expertise*’ is particularly important for ‘*Motivation*’. For different specialties, some competence areas were newly added to the competences needed for the respective specialty. For surgery, the competence areas ‘*Sensory abilities*’ and ‘*Psychomotor & multitasking abilities*’ were mentioned anew. ‘*Sensory abilities*’ were also newly attributed to radiology. ‘*Mental abilities*’ were mentioned as new competence area for psychiatry and internal medicine, while for anaesthesiology, *'Psychomotor & multitasking abilities*' were newly added.

**Conclusions:**

Advanced students seem to be well aware of basic competences needed for clinical practice. Good consensus between students and physicians was only found for psychiatry-specific competences. Medical schools should support their students in matching their perceptions of competences needed for specific specialties with specialty-specific requirements for a realistic choice of a specialty for postgraduate training.

## Background

After having completed their undergraduate medical studies, graduates should have acquired basic competences that enable them to work independently as physicians [1]. Competences represent the individually developed repertoire of abilities, skills, personality traits, and motivational aspects necessary for successful performance within the medical context [2]. Many countries have defined basic learning objectives for undergraduate education so that their students can achieve this goal [3–6]. Having acquired these basic competences, students should be able to start their postgraduate training in any specialty they like to choose [7]. For the work as resident, competences like, *prioritizing work according to clinical urgency* or *responding to individual patients’ health needs* are of particular importance in order to accomplish various medical roles according to the CanMEDS framework for postgraduate education [8]. During postgraduate training, a physician builds on the basic competences acquired in medical school to obtain and develop the specialty-specific competences required for practice in the respective specialty [8].

Medical specialties are characterized by a great diversity in their work requirements, which are associated with different specialty-specific competence profiles as defined by the Requirement-Tracking questionnaire (R-Track) [9]. Very detailed profiles have been described with the R-Track for anaesthesiology [10] and nephrology [11]. Psychomotor and multitasking abilities are particularly needed for specialties with surgical activities, while social interactive competences are of prominent importance for specialties with an intense level of patient-physician-interaction, for example, psychiatry or internal medicine [9]. With respect to the specific competence requirements and a great variety of medical specialties, choosing a medical specialty for residency training seems to be a difficult task for medical students, because the choice usually represents a lifelong career decision [12]. The final year of undergraduate medical education or internship where students get to know different specialties more intensely provides a good opportunity to explore career options [13]. These experiences or working in a particular medical specialty or learning from role models can help students in their decision to choose a specialty for residency [14–16].

Algorithm-based matching programs are also employed to bring applicants and vacancies together [17]. Their aim is to provide realistic information about the specialties and to identify applicants who would be a particularly good fit [18]. Besides interviews with the candidates, the selection process is mainly based on objective criteria such as assessment scores and academic performance [19]. Other aspects like personality [20] or assessment of psychomotor skills for surgical activities [21–23] have also been used for applicant selection. The students’ reasons for choosing a medical specialty are complex and diverse. They can be based on the students’ personality [24, 25], specialty related anticipations such as prestige and income [26] or gender-specific career and lifestyle ideas [27] and anticipated work-life balance of different specialties [28–30]. When applying for a residency position, graduates should have a solid understanding of the required competences that are needed in the different medical specialties. Whether medical students have a realistic perspective on competences required for different medical specialties is not known. This study aims to identify final-year students’ perceptions of basic medical and specialty-specific competences. Comparing the students’ perspectives on medical competences with physicians’ assessment of competences that are required for different medical specialties [9] will provide information whether medical students have a realistic perception of competences that are required for postgraduate training in different specialties. This study’s findings will provide insights whether further competence-based guidance for medical students’ specialty choice for postgraduate education is needed.

## Methods

### Study design and participants

In December 2020, sixty-four advanced medical students from year 4 and 5 of a 6-year undergraduate medical curriculum, 65.6% female and 34.4% male, participated in a competence-based training simulating the first day of residency under pandemic conditions [31]. This training included a telemedicine-based consulting hour with four simulated patients, documentation, and management with electronic patient charts, and one case presentation per participant in a virtual round with an attending physician. Participation was on a voluntary and a first come, first served basis. Eight focus group interviews with eight participants each were conducted directly after the training using a semi-structured interview guideline to identify students’ perceptions of basic medial competences and specific competences needed in different medical disciplines. The study was performed in accordance with the Declaration of Helsinki and the Ethics Committee of the Chamber of Physicians, Hamburg, approved this study and confirmed its innocuousness (PV3649). All participants provided informed written consent for participation in this study. All data were anonymized.

### Interview guideline and interview conduction

The semi-structured interview guideline was developed based on catalogues of basic medical competences [4, 8] and studies regarding basic medical competences [1, 32, 33] and competence profiles of medical specialties [9, 34]. The interview guideline included a brief introduction about the context of the competence-based training the participants had just completed, questions on skills and abilities needed for general medical task, for example, patient consultations, diagnostics, and case presentations, and specific abilities needed in different medical specialties, i.e., anaesthesiology, internal medicine, psychiatry, radiology, and surgery. These specialties were selected as prototypes because they showed significant differences in their competence profiles [9]. With this study, we wished to elucidate whether medical students’ have a realistic perception of and perspective on the different competence profiles of these specialties. All focus group interviews were conducted by E.Z., videotaped, and transcribed verbatim following simple transcription rules which slightly smoothen speech to focus on content [35]. Interviews were anonymized during transcription. The forward translation of exemplary quotes was performed by SH, a physician who holds a C2 level certificate in English language and worked for several years in a hospital in the United States. The back translation was carried out by EZ, a sociologist who has been working in the field of medicine for three years. The translations were checked by VO, a psychologist who has been working in the field of medicine for more than a decade.

### Data analysis

We analysed the transcripts with MAXQDA 2020 (Verbi GmbH) using Braun & Clarke’s thematic analysis, a qualitative method for identifying, analysing, and reporting patterns and themes within data [36, 37]. We developed a detailed overall description of the dataset and used the semantic approach focusing on the identification of explicit meanings of the data, following a realistic paradigm. The thematic analysis included the following six steps: 1) familiarization with the data, 2) generating initial codes, 3) searching for themes, 4) reviewing themes, 5) defining and naming themes, and 6) producing the report [36]. We inductively generated initial codes and searched for themes and deductively assigned themes with respect to competences and competence areas of the Requirement-Tracking questionnaire (R-Track). It includes 63 items that can be assigned to six competence areas: 1) ‘*Personality traits*’, which includes factors that influence the way someone thinks and acts, 2) ‘*Social interactive competences*’, which consists of skills that involve the way someone communicates, interacts and collaborates with others in a team, 3) ‘*Mental abilities*’, consisting of factors that make up cognitive performance, 4) ‘*Sensory abilities*’, which includes factors influencing the perception of the environment, 5) ‘*Psychomotor & multitasking abilities*’, including factors influencing performance in manual control tasks, and 6) ‘*Motivation*’, consisting of factors measuring goal directed effort that leads to performance and expertise [38]. We chose the R-Track for analysis because it is based on the Fleishman Job Analysis Survey (F-JAS) [39] which can be used to assess skills and abilities for different professions [40]. The R-Track was originally adapted from the F-JAS to identify competence profiles of airline pilots and eventually further adapted for health care professionals [38]. This allows for classification of physician competence profiles in specific, as well as larger, professional contexts.

## Results

### Basic medical competences

A total of 220 codes were assigned as aspects of basic medical competences. They could be allocated to 21 Requirement-Tracking questionnaire items, i.e. 33.3% of the 63 R-Track items. These items were represented in four of the six R-Track competence areas (Table 1). Skills and abilities belonging to the area ‘*Social interactive competences*’ were mentioned most frequently (*n* = 113), followed by ‘*Mental abilities*’ (*n* = 39), ‘*Personality traits*’ (*n* = 37), and ‘*Motivation*’ (*n* = 31). No aspects were mentioned from the competence areas ‘*Sensory abilities’* and ‘*Psychomotor & multitasking abilities*’. The four R-Track competence areas, their identified items and sub-themes are presented in Table 2 and illustrated with examples for an extended overview.

**Table 1:** Competence areas and items

Competence area (n)	Items (n)
Social interactive competences (113)	Structuring information (78)
	Tactfulness (15)
	Stress resistance (8)
	Norms & values orientation (4)
	Orientation towards patients (2)
	Coordination & decision making (2)
	Delegation / Delegating (1)
	Persuasiveness (1)
	Sovereignty (1)
	Resistance to monotony (1)
	Coaching & mentoring (0)
	Conflict management (0)
	Diplomacy (0)
	In need of harmony (0)
	Manners & common decency (0)
	Presentation (0)
	Sanctioning (0)
	Self-confidence (0)
	Sense of humour (0)
	Sociability (0)
	Willingness to help (0)
Mental abilities (39)	Concentration (34)
	Clarity of speech (3)
	Memory capacity (2)
	Facility for languages (0)
	Logical reasoning (0)
	Mathematical reasoning (0)
	Numeracy (0)
	Problem comprehension (0)
	Reading comprehension (0)
	Spatial orientation (0)
	Spatial visualization (0)
	Written expression (0)
	Verbal expression (0)
	Verbal understanding (0)
Personality traits (37)	Honesty (24)
	Openness to novelty (9)
	Flexibility (2)
	Prudence (1)
	Cooperation / Agreeableness (1)
	Creativity (0)
	Emotional stability (0)
	Independence and autonomy (0)
	Modesty (0)
	Openness to other people/cultures (0)
	Risk orientation (0)
	Tolerance to frustration (0)
Motivation (31)	Expertise (28)
	Thoroughness (2)
	Endurance (1)
	Achievement motivation (0)
	Reliability & discipline (0)
Psychomotor & multitasking abilities (0)	Multitasking capacity (0)
	Psychomotor coordination (0)
Sensory abilities (0)	Auditory discrimination (0)
	Comprehension (0)
	Hearing sensitivity (0)
	Near vision (0)
	Perceptual range (0)
	Perceptual speed (0)
	Range of field vision (0)
	Selective attention (0)
	Visual imagination (0)

**Table 2:** Exemplary quotations according to competence areas (*n* = 220)

	**R-Track Items** and sub-themes	Exemplary Quotes^a^
**Social interactive competences**	**Structuring information (16)**	“[…] to find […] a structure [in a case] is always important […].”
	Self-organisation (17)	“First of all it is important to structure yourself well […].”
	Selecting information (12)	“[…] what is my first impression [… which information] do I actually need.”
	Prioritising information (11)	“[…] that you first prioritise what is the most important [information].”
	Weighting information (10)	“[…] one can […] assess lab results well [… and] knows […] ‘[this] is acute or […] not […] so important’.”
	Time management (7)	“[…] time management [… has] priority […].”
	Summarizing information (5)	“[…] to summarize the important information […] for someone who has not seen the patient […].”
	**Tactfulness (8)**	"[…] empathy is always helpful […].”
	Change of perspective (7)	"[…] adaptation to the patient[‘s perspective] […].”
	**Stress resistance (4)**	“Stress resistance in any case […].”
	Staying calm (4)	“Staying calm is […] a competence one should have or develop […].”
	**Norms & values orientation (4)**	“[… to have a] clear scheme […].”
	**Orientation towards patients (2)**	“[…] to have enough time for the patient […].”
	**Coordination & decision making (2)**	“[…] and then you have to [decide to] act quickly […].”
	**Delegation / Delegating (1)**	“[…] that you can delegate in things […].”
	**Persuasiveness (1)**	“Being very clear in the way one communicates […].”
	**Sovereignty (1)**	“[…] doing the things one has to do oneself.”
	**Resistance to monotony (1)**	“[…] with the fourth [patient] one thinks ‘[…] the same questions again…’ [… and] one has to [think]: ‘[…] with the next patient I have to be just as attentive’.”
**Mental abilities**	**Concentration (1)**	“[…] I was [only] fully concentrated with the first patient.”
	Focusing (22)	“[…] to be focused all the time […].”
	Attentiveness (7)	“[…] to somehow stay attentive […].”
	Mindfulness (4)	“[…] taking up […] information […] between the lines […].”
	**Clarity of speech (3)**	“[…] to present the patient accurately in a few words […].”
	**Memory capacity (2)**	“[…] [to] remember what one considered before even if something comes up in between […]."
**Personality Traits**	**Honesty (1)**	“[…] one has to deal with the [different patient] personalities without, [e.g.], raising false hope […].”
	Being unprejudiced (7)	“[…] not letting oneself being guided by [one’s prejudice towards a patient] […].”
	Self-reflection (7)	“[…] you also must be able to reflect on yourself […].”
	Dealing with ignorance (5)	“[…] but also, [that] you are not afraid to say ‘I don't know’ […].”
	Asking for help (3)	“[…] that one is not afraid to say […] ‘can you help me, please’.”
	Transparency (1)	“[…] being very clear in the way one communicates.”
	**Openness towards novelty (9)**	“[…] make sure [to] take other things into account […].”
	**Flexibility (2)**	“[…] flexibility [to] adapt to the situation […].”
	**Prudence (1)**	“[…] patience […] we do this and that and wait for the results […].”
	**Cooperation / Agreeableness (1)**	“[…] good communication and interaction in the team […].”
**Motivation**	**Expertise (5)**	“[…] broad knowledge, specialist knowledge [… to] know […] which differential diagnoses exist.”
	Communication techniques (15)	“[…] structured conversation is […] important.”
	Pattern recognition (4)	“[…] you have to know the symptoms and the associated diseases […].”
	Technical skills (3)	“[…] one has to be able to deal with technology […].”
	Abbreviations (1)	“[….] to know all the abbreviations [… for] fast documentation […].”
	**Thoroughness (2)**	“[…to make sure] that everything is complete [and] nothing is missing […].”
	**Endurance (1)**	“Definitely endurance, I would say […].”

^a^All exemplary quotes are samples from our survey

### Social interactive competences

The aspects assigned to the competence area ‘*Social interactive competences*’ covered 47.6% of its 21 R-Track items. Within the item ‘*Structuring information*’, 16 aspects could be directly assigned, and six sub-themes were discovered: ‘*Self-organisation*’, ‘*Selection information’*, ‘*Prioritising information*’, ‘*Weighting information*’, ‘*Time management*’, and ‘*Summarizing information*’. The item ‘*Tactfulness*’ included only the sub-theme ‘*Change of perspective’*. ‘*Staying calm*’ was discovered as a sub-theme of the item ‘*Stress resistance*’. Further aspects mentioned by the students could be assigned to the items ‘*Norms & rule orientation*’, ‘*Orientation toward patients*’, ‘*Coordination & decision making*’, ‘*Delegation / Delegating*’, ‘*Persuasiveness*’, ‘*Sovereignty*’, and ‘*Resistance to monotony*’.

### Mental abilities

The aspects assigned to the competence area ‘*Mental abilities*’ covered 21.4% of its 14 R-Track items. Within the item ‘*Concentration*’, three sub-themes were discovered: ‘*Focusing*’, ‘*Attentiveness*’, and ‘*Mindfulness*’. Further aspects mentioned by the students could be assigned to the items ‘*Clarity of speech*’ and ‘*Memory capacity*’.

### Personality traits

The aspects assigned to the competence area ‘*Personality traits*’ covered 41.7% of its 12 R-Track items. The item ‘*Honesty*’ included five sub-themes: ‘*Being unprejudiced*’, ‘*Self-reflection*’, ‘*Dealing with ignorance*’, ‘*Asking for help*’, and ‘*Transparency*’. Further reported items were ‘*Openness to novelty*’, ‘*Flexibility*’, ‘*Prudence*’, and ‘*Cooperation / Agreeableness*’.

### Motivation

The aspects assigned to the competence area ‘*Motivation*’ covered 60% of its 5 R-Track items. The item ‘*Expertise*’ approached with four sub-themes: ‘*Communication techniques*’, ‘*Pattern recognition*’, ‘*Technical skills*’, and ‘1’. Further aspects mentioned by the students could be assigned to the items ‘*Thoroughness*’ and ‘*Endurance*’.

### Specialty-specific competences

**Fig. 1:**
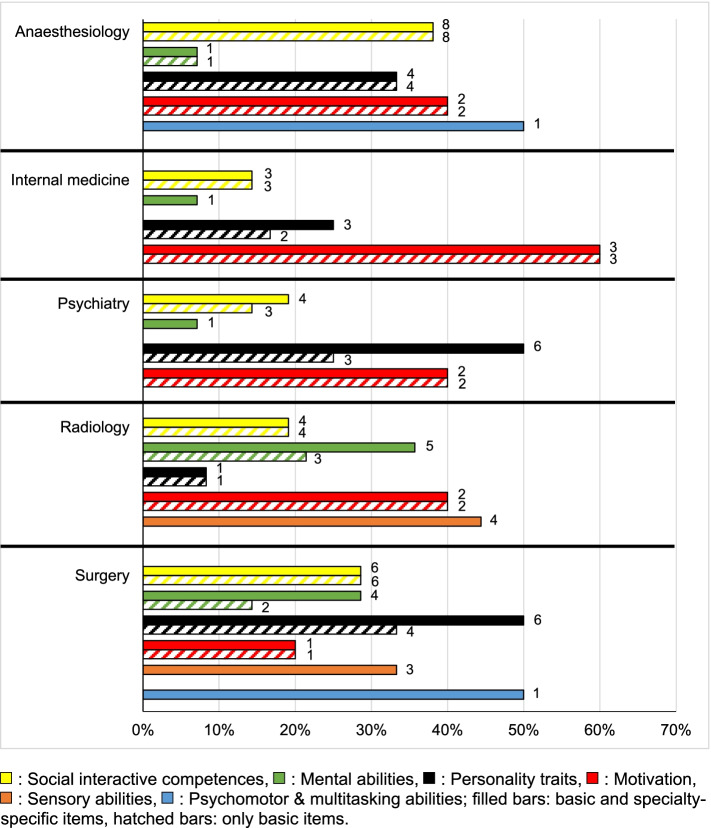
Percentage of R-track items per competence area and specialty

A total of 231 codes were assigned to five different medical specialties: anaesthesiology (*n* = 55), internal medicine (*n* = 42), psychiatry (*n* = 52), radiology (*n* = 39) and surgery (*n* = 43). These included basic competences that were mentioned per specialty and specialty-specific competences. Table 3 shows the newly mentioned specialty-specific aspects at individual item level that were not already discussed as basic competences. While for anaesthesiology only one item from the area ‘*Psychomotor & multitasking abilities*’ was mentioned as being specialty-specific and for internal medicine only one item each from the areas ‘*Mental abilities*’ and ‘*Personality traits*’, many more items from different competence areas were identified as being specialty-specific for psychiatry, radiology, and surgery. Figure 1 shows the percentage of total R-track items mentioned per specialty versus basic items from the six competence areas, which were also mentioned for the respective specialty as being specialty-specific. In the competence area ‘*Social interactive competences*’, specialty-specific competences occurred only for psychiatry (4.8%). In the area ‘*Mental abilities*’, new aspects were mentioned for internal medicine (7.1%), psychiatry (7.1%), radiology (21.4%), surgery (14.6%). In the area ‘*Personality traits*’, specialty-specific competences occurred for internal medicine (8.3%), psychiatry (25%) and surgery (16.7%). No additional aspects were mentioned for any specialty regarding the area ‘*Motivation*’. Specialty-specific aspects from the area ‘*Sensory abilities*’ included only new aspects and occurred only for radiology (44.4%) and surgery (33.3%). With regard to the area ‘*Psychomotor & multitasking abilities*’, specialty-specific aspects were only mentioned for anaesthesiology (50%) and surgery (50%).

**Table 3:** Specialty-specific items according to competence areas

	**R-Track items**	**A *n* = 2**	**I *n* = 2**	**P *n* = 21**	**R *n* = 11**	**S *n* = 13**
**Social interactive competences**	**Sanctioning**			**1**		
**Mental abilities**	**Written expression**		**1**		**3**	
	**Verbal expression**				**2**	
	**Problem comprehension**			**1**		**2**
	**Spatial visualization**					**1**
**Personality traits**	**Emotional stability**		**1**	**11**		**1**
	**Openness to other people**			**7**		
	**Modesty**			**2**		**1**
**Sensory abilities**	**Perceptual range**				**2**	**2**
	**Perceptual speed**				**2**	**1**
	**Comprehension**				**1**	**1**
	**Near vision**				**1**	
**Psychomotor & multitasking abilities**	**Psychomotor coordination**	**2**				**4**

*A* Anaesthesiology, *I* Internal Medicine, *P* Psychiatry, *R* Radiology, *S* Surgery

## Discussion

Medical students recognized many essential aspects related to basic competences needed by physicians. The highest number of aspects was found in the competence area of ‘*Social interactive competences*’ which resembles a core component of undergraduate medical education [41]. The students mentioned, for instance, ‘*Structuring information’*, ‘*Tactfulness*’ and ‘*Stress resistance*’ from this competence area. Structuring information about patients is a central aspect of clinical reasoning [42] that constitutes a basic competence for all specialties, exemplary shown for internal medicine [43] or orthopaedics [44]. Tactfulness is of great importance in patient-physician interaction and an essential component of medical professionalism [45]. Stress resistance is an important aspect for health professionals because their work is often associated with high levels of stress [46] which can have a negative impact on professional performance and quality of patient care [47, 48]. As a basic mental skill, students emphasized the ability to concentrate, which has been shown to be closely linked to clinical decision-making [49] and has been shown in surgery to be needed to execute difficult manual work [50]. ‘*Honesty*’ was a particularly important personality trait for physicians in general from the students’ perspective. The patient-physician relationship constitutes a special interpersonal relationship based on honest information about the diagnosis and the outcome [51]. Students were also aware that medical expertise is particularly important for motivation. Competency profiles of different medical specialties showed that ‘*Motivation*’ was the highest rated competence area in almost all specialties [9]. Motivation is a fundamental aspect for the profession of medicine, which is also decisive for the choice of a speciality. In the context of choosing a specialty, it is particularly interesting to see whether the students' ideas match those of the specialties.

For the five investigated specialties, the students mentioned at least one aspect from a competence area that had not been mentioned for the respective specialty as basic competence.

Surgery showed the greatest differences between basic and specialty-specific competences. Aspects from the two competence areas ‘*Sensory abilities*’ and ‘*Psychomotor & multitasking abilities*’ were only mentioned as being specialty-specific for surgery. ‘*Psychomotor coordination*’ is acquired in postgraduate training, for example, with laparoscopic or arthroscopic simulators [52, 53]. As additional surgery-specific aspect informs the competence area ‘*Mental abilities*’ ‘*Problem comprehension*’ was mentioned, which is required when selecting patients for surgical treatment [54]. ‘*Emotional stability*’ was additionally addressed as an exemplary aspect of ‘*Personality traits*’, which has been shown to be higher in surgeons than in the population norms [55]. The competence area ‘*Sensory abilities*’ was newly added by our participating students as being specialty-specific for radiology and included the aspects ‘*Perceptual range*’ and ‘*Perceptual speed*’ which can be measured with radiology-specific tests [56]. Several aspects from the competence area ‘*Mental abilities*’ were mentioned as specialty-specific aspects of radiology, for example, ‘*Written expression*’. Indeed, the written radiology report is a key component in the communication between radiologists and referring physicians [57]. ‘*Mental abilities*’ emerged as new specialty-specific competence area for psychiatry with ‘*Problem comprehension’* being a relevant aspect. In residency training, problem-based conferences were a successful teaching method for psychiatry residents to acquire psychiatry patient management [58]. From the competence area ‘*Personality traits*’, students particularly mentioned ‘*Emotional stability*’ and ‘*Openness to other people*’ as specialty-specific for psychiatry. In personality analyses of physicians from different specialties, psychiatrists have been found to reach high scores for ‘*Emotional stability*’ [59] and ‘*Openness*’ [60]. ‘*Mental ability*’ was also a newly mentioned competence area for the specialty of internal medicine with the aspect ‘*Written expression*’, which can be trained during internal medicine residency by the scholarly activity of writing case reports [61]. ‘*Emotional stability*’, was identified as an internal medicine-specific aspect in the competence area ‘*Personality traits*’ and seems to be highly necessary, since 76% of internal medicine residents met the criteria for burnout [62], ‘*Psychomotor coordination*’ was mentioned by the students as the only new specialty-specific aspect from the additional specialty-specific competence area ‘*Psychomotor & multitasking abilities*’ for anaesthesiologists. Indeed, good manual movement and hand–eye coordination is necessary for anaesthesiologists to perform complex psychomotor tasks such as placing a nasotracheal intubation [63]. Overall, the students had a good perception of the competences needed for different specialties as assessed by physicians from the respective specialties [9]. The best match was found for psychiatry. For surgery and radiology, the students overestimated the relevance of ‘*Sensory abilities*’ and they underestimated it for anaesthesiology. They also overestimated ‘*Social interactive competences*’ for anaesthesiology while they underestimated these for internal medicine. The students somewhat underestimated ‘*Motivation*’ for surgery and seem to have overestimated ‘*Personality traits*’ a bit for this specialty.

A limitation of our study was that the respondents only came from one medical school. Since their participation in the simulation was voluntary, self-selection could have led to particularly interested and engaged participants. Furthermore, we did not distinguish between male and female participants in the focus groups which could have led to somewhat distorted results. However, the distribution of male and female participants resembled the distribution among medical students in general at our medical school. Interestingly, 42 of the 63 competences were not mentioned by the students. These include mostly general competences like ‘Comprehension’, ‘Memory capacity’, or ‘Sociability’. Since we did not specifically discuss competences with the students that were not mentioned with respect to their relevance for physicians or specific specialties it remains unknown, whether students took them for granted or regarded them as irrelevant. This needs to be addressed in further studies. A strength of this study are the semi-structured interviews conducted immediately after the training. The simulation experience made it easier for students to visualize the competences they needed for independent medical practice rather than thinking of their abstract definitions. The data collection in association with the training allowed participants to talk openly about their experiences while being guided thematically by the interviewer. With this qualitative approach, we have provided a first insight into the perceptions of advanced medical students on required basic and specialty-specific competences. A closer look at the specialties of anaesthesiology, internal medicine, psychiatry, radiology, and surgery showed that the students already had quite good perceptions of basic competences, but there were still some inconsistencies with regard to the specialty-specific competences. Students should compare their ideas about a specialty they would like to choose for postgraduate training with the competence profile suggested by physicians from the respective specialty. This could lead to a more realistic picture of specialty-specific competence requirements and eventually prevent dropouts of postgraduate training. Additionally, medical educators could provide specialty-specific training for undergraduate students in clerkships for competence areas which are specifically required by a specific specialty.

## Conclusions

The medical students in this study seem to have developed a good perception of the necessary basic competences for clinical practice. With regard to the specific competence requirements of different disciplines, a high degree of agreement on specialty-specific competences between students and physicians was only found for psychiatry, while a lack of consensus with regard to specialty-specific competences remained for anaesthesiology, internal medicine, surgery, and radiology. Incorrect perceptions of specialty-specific competences could lead to wrong concepts about what to expect of residency training in the respective specialty. Students should be invited to compare their ideas of specialty-specific competence profiles with the competence requirements as assessed by physicians from a respective specialty to get a realistic impression of specialty–specific postgraduate training. Courses during undergraduate education in specialty-specific competences could also prepare students to develop a realistic impression of the different competence profiles of medical specialties and support their choice of specialty for residency training.

## Data Availability

All data and materials are available from the manuscript.

## Abbreviations

CanMEDS: Canadian medical education directives for specialists
R-Track questionnaire: Requirement-Tracking questionnaire
